# Radiofrequency ablation for pediatric recurrent hepatocellular carcinoma: a single-center experience

**DOI:** 10.1186/s12880-023-01159-3

**Published:** 2023-12-06

**Authors:** Haiyi Long, Wenxin Wu, Luyao Zhou, Hui Shen, Xiaoyan Xie, Baoxian Liu

**Affiliations:** Department of Medical Ultrasonics, Institute of Diagnostic and Interventional Ultrasound, The First Affiliated Hospital of Sun Yat-sen University, 58 Zhongshan Road 2, Guangzhou, 510080 China

**Keywords:** Radiofrequency ablation, Recurrent hepatocellular carcinoma, Children, Safety, Local tumor progression, Survival

## Abstract

**Purpose:**

To summarize our single-center experience with percutaneous ultrasound (US)-guided radiofrequency ablation (RFA) for pediatric recurrent hepatocellular carcinoma (RHCC).

**Methods:**

From September 2007 to September 2021, patients under 18 who underwent percutaneous US-guided RFA for RHCC were retrospectively enrolled in this study. Local effectiveness, complications, local tumor progression (LTP), progression free survival (PFS), and overall survival (OS) were evaluated.

**Results:**

A total of 10 patients (9 male and 1 female; mean age, 11.7 ± 4 years ; age range, 6–17 years) with 15 intrahepatic RHCC lesions were enrolled in this study. Complete ablation (CA) was achieved in 14 out of 15 lesions (93.3%) after the first RFA. During the follow-up (mean, 63.1 ± 18 months; range, 5.3-123.3 months), LTP did not occur. Five patients died including three with tumor progression and one with liver failure. The accumulative one- and three-year PFS rates were 30% and 10%, respectively. The accumulative one- and three-year OS rates were 77.8% and 44.4%, respectively.

**Conclusions:**

Our single-center experience suggests the safety and feasibility of percutaneous US-guided RFA for pediatric RHCC.

## Introduction

Hepatocellular carcinoma (HCC) is a rare liver malignancy in children that accounts for less than 1% of all pediatric malignancy [1]. The prognosis of pediatric HCC remains poor, with a reported 5-year survival rate of 20–30% in most studies [2, 3]. As demonstrated, high frequency of recurrence is the major cause of poor outcomes for pediatric HCC [4]. According to the guideline of Childhood Liver Cancer Treatment (PDQ), for those with isolated recurrence in the liver, chemoembolization temporization before transplant or immediate liver transplant (LT) is suggested. Otherwise, Phase I/II clinical trials should be considered [5]. However, the application of LT is challenging due to shortage of donor, and extensive hilar adhesions and high risk of infective complications in the setting of immunosuppression after LT for the initial HCC. Therefore, more treatment modalities for recurrent HCC (RHCC) in children are warranted.

Percutaneous radiofrequency ablation (RFA) is recommended as the first-line treatment modality for early-stage HCC with effective local tumor control and survival benefit in adult patients [6]. RFA also shows non-inferior performance when compared with repeated liver resection for RHCC, especially in those with recurrence smaller than 5 cm [7, 8]. Considering the growth and development of children, percutaneous ultrasound (US)-guided RFA could be considered as a treatment option for the eradication of pediatric liver malignancy due to its minimal invasiveness and absence of drug or radiation toxicity. The potential value of RFA for pediatric liver masses has been reported in previous studies [9–12]. Yet, few studies have discussed the utility of percutaneous RFA for RHCC in children. Therefore, the aim of current study was to summarize our single-center experience of percutaneous US-guided RFA for RHCC in children.

## Materials and methods

### Patients

This retrospective study was performed to review patients under age 18 who received percutaneous US-guided RFA for intrahepatic RHCC from September 2007 to September 2021 from the database from our department. A total of 10 patients were enrolled. The diagnosis of RHCC was based on the following criteria: (1) an initial diagnosis of HCC confirmed by histopathology; (2) newly-detected intrahepatic lesions that that met diagnostic criteria of HCC according to the American Association for the Study of Liver Diseases (AASLD) guideline during postoperative follow up [13], and confirmed by histopathology obtained from liver biopsy before the RFA procedure. The inclusion criteria were as follows: (1) aged under 18 years; (2) a history of liver resection or LT for initial HCC confirmed by histopathology; (3) newly-detected intrahepatic lesions that met diagnostic criteria for HCC according to the AASLD guideline [13]; (4) for curative intent, defined as patients with limited intrahepatic recurrence (solitary lesion with a diameter ≤ 5 cm, or 3 or fewer lesions with a diameter ≤ 3 cm for each) which could be ablated simultaneously; for palliative intent, defined as patients with multiple intrahepatic recurrence with a diameter > 3 cm, or macrovascular invasion or extrahepatic metastases detected by imaging; (5) liver function with Child-Pugh grade A or B; (6) East Coast Oncology Group (ECOG) performance status score of 0 or 1 [14]. Exclusion criteria were as follows: severe coagulopathy, cardiopulmonary dysfunction, or infection.

### Data collection

Baseline characteristics of patients were collected including age, gender, initial treatment of HCC, purpose of RFA. Laboratory examination were collected within one week before the application of RFA, including: (1) complete blood count (count of platelet, white blood cell, etc.); (2) liver function test (albumin, alanine aminotransferase, aspartate aminotransferase, total bilirubin); (3) hepatitis (serum hepatitis B surface antigen, serum hepatitis B surface antibody, serum hepatitis B envelop antigen, serum hepatitis B envelop surface antibody, serum hepatitis B core antibody and hepatitis C virus antibody); (4) serum alpha-fetoprotein (AFP). Contrast-enhanced ultrasound (CEUS), contrast-enhanced computed tomography (CECT), or contrast-enhanced magnetic resonance imaging (CEMRI) were performed to evaluate intrahepatic lesions including size, number, location, and relationship with adjacent organs. The Child-Pugh score and albumin-bilirubin (ALBI) score were calculated.

### RFA procedure

**Fig. 1:**
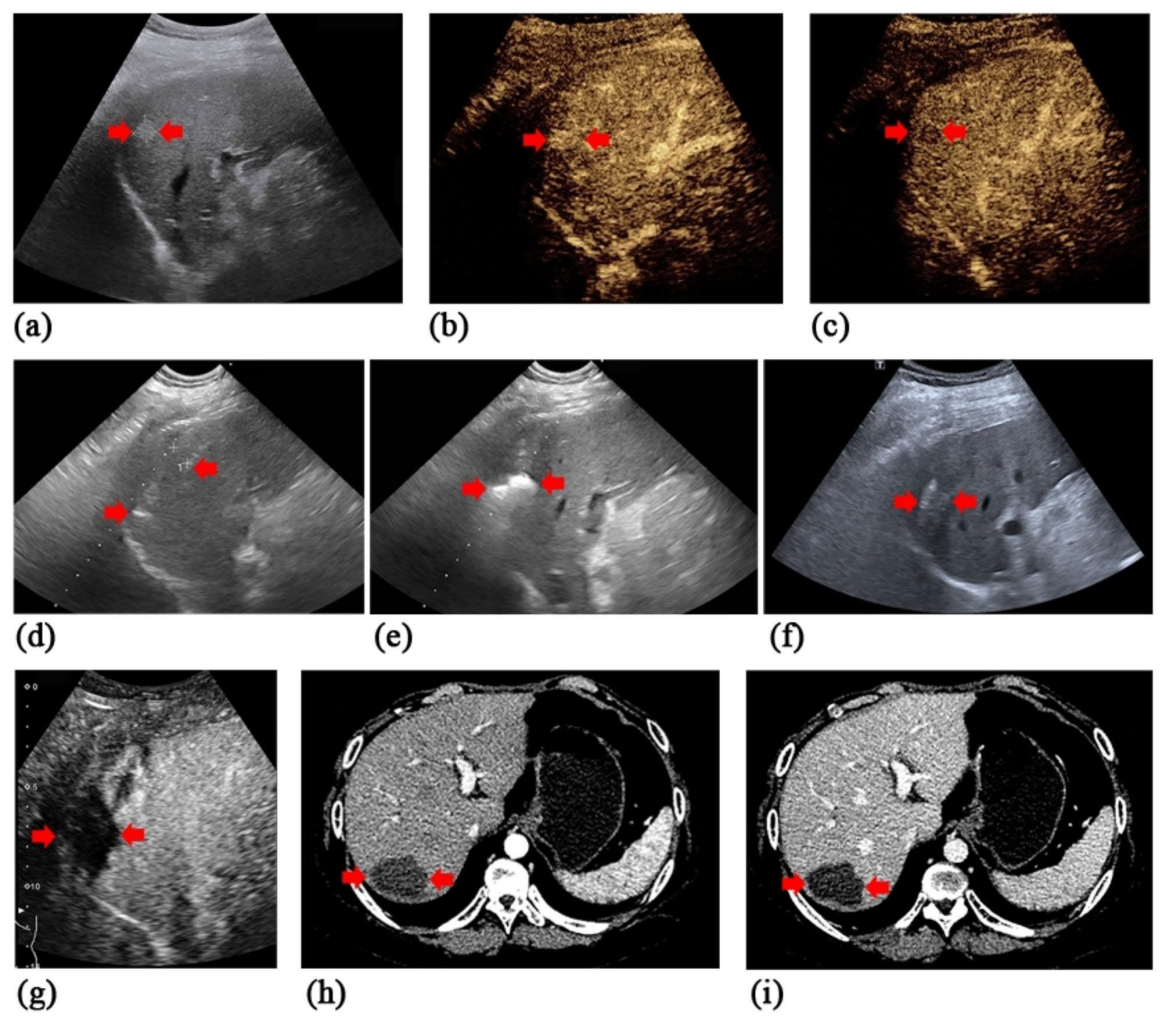
A 14-year-old male was diagnosed as intrahepatic recurrent hepatocellular carcinoma (RHCC) at 7 months after initial liver transplantation (LT). Ultrasound-guided radiofrequency ablation (RFA) was performed. (**a**) A hyperechoic lesion located in Segment 7, with a size of 1.4cmx1.2cmx1.2cm was detected by the grayscale ultrasound. (**b, c**) On the contrast-enhanced ultrasound (CEUS) images, the targeted lesion displayed homogeneous hyperenhancement in the arterial phase (b) and washout in the portal phase (c). (**d**) Under the guidance of real-time ultrasound, the No-touch technique was performed by sequentially inserting two electrodes around the periphery of the target lesion and activating them alternatively to perform ablation with a sufficient peri-tumoural margin and avoid direct puncture of the tumor. The second radiofrequency electrode was being inserted, with an inter-electrode distance of 1.1 cm. (**e**) Hyperechoic zone around the electrode tips appeared and the range extended as the RFA procedure proceeded. (**f, g**) The next day after RFA, the ablative zone exhibited as heterogeneous hyperechoic on the grayscale ultrasound (f) and displayed no enhancement in all phases on the CEUS images, indicating a complete ablation (g). (**h, i**) Two months after RFA, the target lesion exhibited non-enhancement on the contrast-enhanced CT (CECT) images

Percutaneous RFA procedures were performed under the guidance of real-time US by a single experienced operator (X.X.Y and X.X.H with over 20 years of experience with US). When the target lesion was opaque on conventional US, CEUS or fusion imaging of real-time US and CT images was adopted for guidance [15] (Fig. 1, a-c). RFA was carried out with intravenous anesthesia with vital signs continuously monitored by anesthesiologist. Using US surveillance, the radiofrequency electrode (Cool-tip™, Valleylab, Boulder, CO, USA) was carefully inserted into or adjacent to the target lesion (Fig. 1, d-e). The number of electrodes was dependent on the tumor size, shape, and location with the aim of reaching a minimum safety margin of 0.5 cm. In general, lesion with a maximal diameter of 1.5 cm could be treated with one single electrode. For a larger lesion, or lesion in which the ‘no-touch’ RFA was performed, two or more electrodes were adopted [16]. When necessary, combination with percutaneous ethanol injection (PEI) was adopted in high-risk lesions defined as tumors within 5 mm of the capsule (subcapsular), a vital structure, or a blood vessel 3 mm in diameter or larger [17]. After completion of the ablation, the electrode was retracted along the needle track carefully by 1 cm increments to prevent bleeding and tumor seeding. Complications were reported using the Dino-Clavien classifications [18].

### Follow up

One month after RFA, CEUS or CECT was performed to evaluate local efficacy. Afterward, imaging examinations (CEUS, CECT or CEMRI) as well as laboratory tests (liver function and AFP) were taken every three months during the first 2 years, and every 4–6 months thereafter.

Complete ablation (CA) was defined as non-enhancement of the ablated zone within one month (Fig. 1, f-i), otherwise it was considered as incomplete ablation (ICA) [19]. For ICA, additional ablation was performed until reaching CA. Local tumor progression (LTP) was defined as enhancement reappearance of tumor tissue within or adjacent to the ablation site after reaching CA [20]. Progression free survival (PFS) was defined as the time from the first RFA for RHCC to intrahepatic recurrence, extrahepatic metastasis, death, or last follow-up. Overall survival (OS) was defined as the time from the first RFA to death or last follow-up. When recurrence was detected during the follow-up, repeated RFA was offered if technically feasible, otherwise corresponding treatments were given according to the tumor characteristics, liver function and the requests of patient [19]. ICA and LTP were evaluated on a tumor-by-tumor basis while PFS and OS were evaluated on a patient-by-patient basis.

### Statistical analysis

All statistical analyses were carried out using SPSS (SPSS Statistics, Version 19.0; IBM, Armonk, NY). Continuous variables were presented as means ± standard deviation and analyzed using the Student’s t test or the Mann-Whitney test. Categorical variables were presented as numbers and proportions and analyzed using the Pearson χ2 or Fisher exact test. Cumulative LTP and survival rates were estimated using the Kaplan-Meier method. A two-tailed P value < 0.05 was considered significant.

## Results

### Baseline clinical characteristics

The baseline characteristics of the patients were summarized in Table 1. In total, 10 patients (9 male and 1 female) with 15 intrahepatic RHCC lesions were enrolled in this study. The mean age was 11.7 ± 4 years (range, 6–17 years). Three patients with extrahepatic metastasis received RFA as palliative care, including two patients with pulmonary metastasis and one patient with both pulmonary and splenic metastasis. The majority (n = 8) had hepatitis B virus infection. Most of the patients (n = 9) were classified as Child-Pugh grade A.

**Table 1:** Baseline characteristics of the patients

Characteristics	n = 10
Age (year) *	11.7 ± 4 (6–17)
Gender (Male/Female)	9/1
Purpose of RFA (Curative/Palliative)	7/3
Hepatitis infection (Yes/No)	8/2
Cirrhosis (Yes/No)	6/4
Primary treatment (Resection/Transplantation)	8/2
Child-Pugh grade (A/B)	9/1
ALBI grade (1/2/3)	6/3/1
ECOG PS score (0/1)	7/3
Biochemical examinations	
AFP (ug/L)	7961 (2.8-50260)
ALT (IU/L)	39.1 ± 19 (11–71)
ALB (g/L)	38.9 ± 7.4 (20-45.1)
TBIL (µmol/L)	10 ± 3.5 (6.3–16.7)
PT (s)	12.5 ± 1.4 (10.2–15.3)
WBC account (×10^9^/L)	5.11 ± 2 (3–10)
PLT account (×10^9^/L)	152.6 ± 82 (18–306)
Tumor number (Solitary/Multiple)	5/5

* Expressed in mean ± standard deviation (range), or in median (range). RFA: radiofrequency ablation; ALBI: albumin-bilirubin; ECOG PS score: Eastern Cooperative Oncology Group; AFP: alpha-fetoprotein; ALT: alanine aminotransferase; ALB: albumin; TBIL: total bilirubin; PT: prothrombin time; WBC: white blood cell; PLT: platelets

### Tumor characteristics, RFA strategy and treatment outcomes

The mean size of the tumors was 1.8 ± 0.9 cm (n = 15; range, 1-4.6 cm). Two lesions adjacent to hepatic vein (Segment 2 and Segment 5), two lesions adjacent to Glisson’s capsule (Segment 5), one lesion adjacent to surgical margin (Segment 6), one lesion adjacent to portal vein (Segment 2), and one lesion adjacent to inferior vena cava, portal vein and bile duct (Segment 1), were identified. Among them, RFA combining PEI with a volume of 10ml ethanol was performed in the lesion located in Segment 1 (Table 2). No complications related to RFA occurred during and after the RFA procedure.

**Table 2:** RFA treatment and response

Characteristics	n = 15
Tumor size (cm) *	1.8 ± 0.9 (1-4.6)
High-risk location (Yes/No)	7/8
Combined with PEI (Yes/No)	1/14
Response (CA/ICA)	14/1
LTP (Yes/No)	0/15

* Expressed in mean ± standard deviation (range). RFA: radiofrequency ablation; PEI: percutaneous ethanol injection; CA: complete ablation; ICA: incomplete ablation; LTP: local tumor progression

ICA was identified in one tumor and the other 14 tumors were classified as CA (Fig. 1). The CA rate after the first RFA was 93.3% (14/15). Additional RFA was performed in the ICA lesion, which achieved CA thereafter.

### Follow-up

**Fig. 2:**
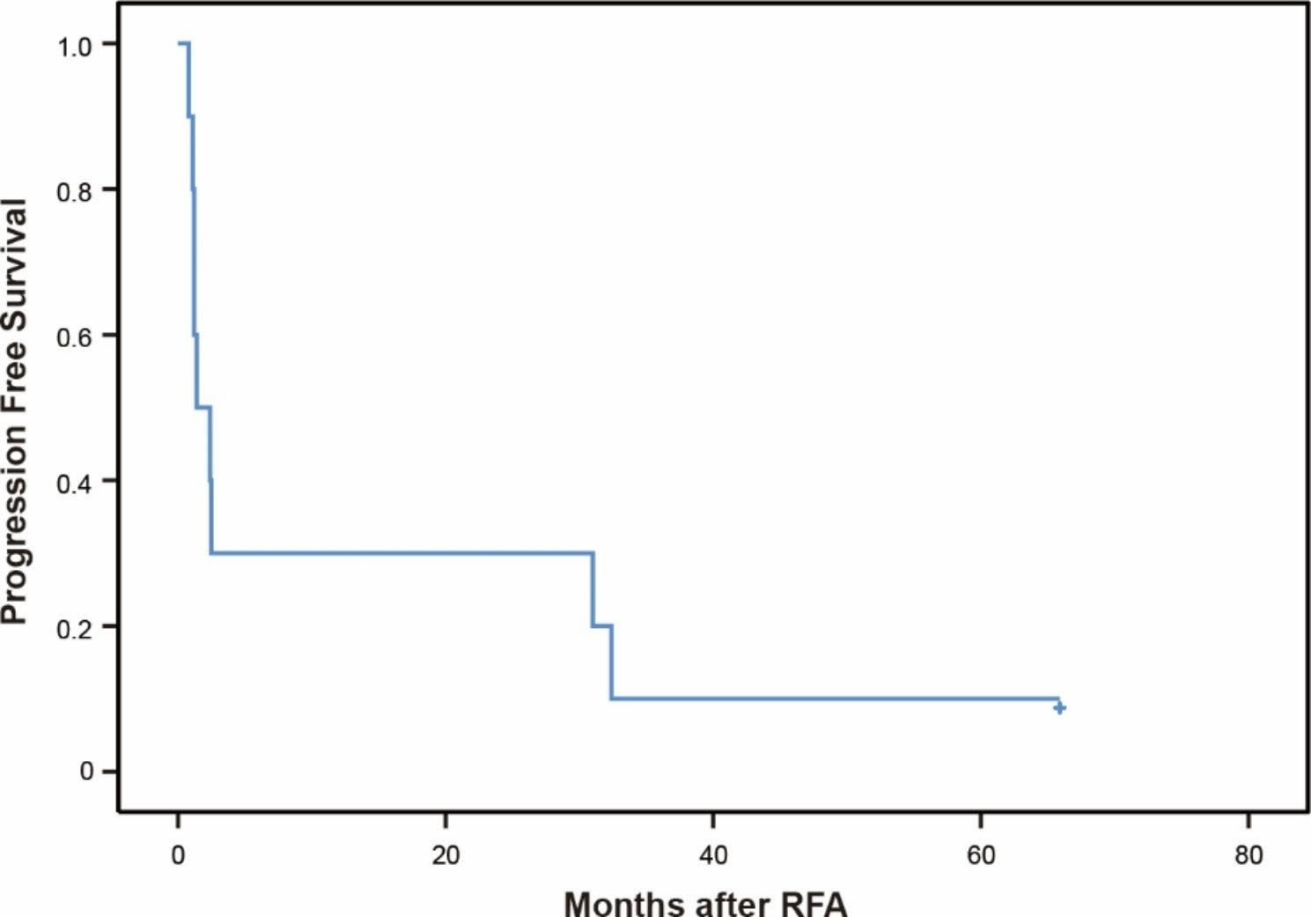
Progression-free survival curve of patients after RFA for intrahepatic RHCC. RFA, radiofrequency ablation; RHCC, recurrent hepatocellular carcinoma

All the patients entered follow-up and the mean follow-up period was 63.1 ± 18 months (range, 5.3-123.3 months). During the follow-up, LTP did not occur in all tumors. Tumor progression was detected in 9 patients (intrahepatic recurrence, n = 3; extrahepatic metastasis, n = 1; intrahepatic recurrence concomitant with extrahepatic metastasis, n = 5). The median PFS time was 1.4 months (95% CI, 0-3.3 months). The cumulative 1- and 3-year PFS rates were 30% and 10%, respectively (Fig. 2).

**Fig. 3:**
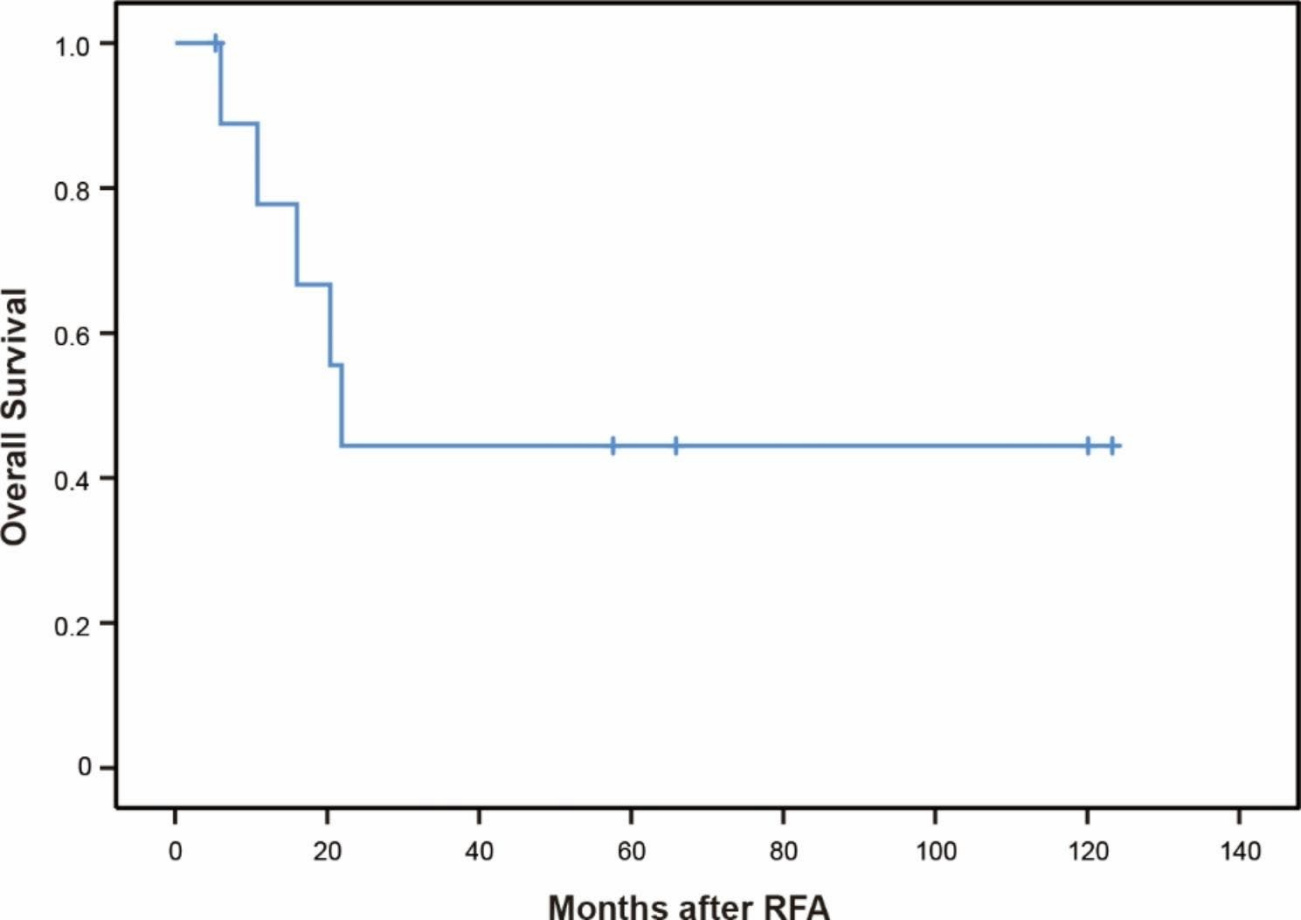
Overall survival curve of patients after RFA for intrahepatic RHCC. RFA, radiofrequency ablation; RHCC, recurrent hepatocellular carcinoma

At the end of follow-up, five patients died and one patient was lost after 5.3 months of follow-up. The causes of death were tumor progression (n = 3), liver failure (n = 1), and pneumonia (n = 1). The median OS time was 21.9 months (95% CI: 17.5–26.3 months). The cumulative 1- and 3-year OS rates were 77.8% and 44.4%, respectively (Fig. 3).

## Discussion

HCC is a rare liver malignancy in children, with less than 1.5 cases per million children under 18 years [1]. Due to innovations of surgical strategies particularly in LT, outcomes of pediatric HCC seem to improve over the decades, with a prolonged 5-year OS rate up to 83% reported in previous studies [21, 22]. However, recurrence remains the major cause of death and the outcomes of RHCC remains dismal. The current study summarized our single-center experience of percutaneous US-guided RFA for RHCC in children.

Pediatric HCC is far less investigated compared to HCC in adults, which might be associated with the extreme low incidence rate. Previous studies indicate that salvage LT is superior to repeated liver resection, especially in terms of disease-free survival for RHCC [23, 24]. For children with isolated recurrence in the liver, chemoembolization temporization before LT or immediate LT were reported, but salvage LT is limited due to a scarcity of donor organs, a high cost, and a long waiting list [5]. Repeated liver resection, especially by laparoscopy, could achieve tumor eradication and provide favorable long-term outcomes [25–27]. However, repeated surgery either salvage LT or re-resection might not be an option for children with RHCC who cannot tolerate surgical stress. Radiotherapy, such as Yttrium-90 transarterial radioembolization (Y90-TARE) and SBRT, has been reported in the treatment of RHCC [28, 29]. For now, few studies have reported the use of Y90-TARE or SBRT in pediatric liver malignancy, which might be associated with radiation exposure, and the need of specialized equipment and technical expertise required for its safe use [30, 31]. TACE is an alternative treatment modality for childhood liver malignancy [32]. However, TACE is a radiation-guided procedure that cannot achieve tumor eradication, which might limit its use in children. Since 1990, the International Childhood Liver Tumor Strategy Group has launched a serial of clinical trials (SIOPELs) to investigate the utility of chemotherapy for pediatric HCC. Although responses to chemotherapy were observed, it failed to improve overall survival of pediatric HCC [2, 3]. In recent years several clinical trials have been launched to investigate novel cytotoxic agents for recurrent or refractory solid tumors in children [33–35]. With only a small proportion of patients with liver malignancy in these studies, the efficacy of these chemical agents could not be determined for pediatric HCC. Moreover, targeted therapy and immunotherapy have offered novel therapeutic opportunities for HCC, but current studies are mostly adult-based [36]. Recently a phase I study combining sorafenib and irinotecan in pediatric refractory liver cancer failed to meet the end point due to severe adverse events [37]. Therefore, further studies are needed to verify the potential value of systemic therapy in pediatric RHCC.

To date, no studies on the efficacy of percutaneous RFA have been conducted in the particular setting of pediatric RHCC. Hetzer et al. reported the utility of percutaneous stereotactic RFA for various liver masses in children [10]. Compared to their study, we adopted US-guided RFA, which was convenient and free of radiation. All tumors achieved CA with a 100% technical success rate and neither severe RFA-related adverse effects nor mortality were seen in our study, which supports the idea that percutaneous RFA is feasible and safe to treat pediatric RHCC.

We applied RFA combined with PEI to one tumor that located in the caudate lobe surrounded by the inferior vena cava, portal vein and bile duct, known as the high-risk location. Our previous studies demonstrated that combination of RFA and PEI for tumors in the caudate lobe can achieve high treatment success rate, but tumor size larger than 2 cm increases the risk of LTP after RFA [38, 39]. The tumor was 4.6 cm in size and received ethanol volume of 10 ml before RFA. Although ICA was identified one month after RFA, the tumor achieved CA after repeated RFA, which indicated that for lesions adjacent to important structures, close follow-up is necessary to identify residual tumor in a timely manner.

In addition, three patients diagnosed as pulmonary metastasis of HCC received RFA treatment with palliative intent in our study. Previous study showed that aggressive management of intrahepatic lesions might provide survival benefits for synchronous HCC with pulmonary metastasis [40]. For patients in advanced stage, palliative treatment may improve outcomes by decreasing tumor burden and relieving symptoms [41]. Among these three patients, one patient received subsequent targeted therapy combined with immunotherapy, and died of pneumonia and asphyxia 20 months after RFA. One patient received subsequent chemotherapy and died of liver failure 10.8 months after RFA. The third patient did not receive treatment and died of tumor progression 6 months after RFA. The prognosis of patients with advanced-stage HCC is poor, and the utility of RFA needs further investigation in this setting.

Our study had some limitations. First, only 10 patients with RHCC after curative surgery who underwent percutaneous US-guided RFA were enrolled in this study. However, pediatric HCC is extremely rare and thus, it is difficult to collect a large sample size of patients. Secondly, retrospective data collected from a single institution might result in bias. Therefore, a properly designed prospective multicenter study is needed to determine the efficacy of RFA in this setting. Finally, there was no direct comparison to other treatment modalities in this single arm treatment study. Randomized controlled clinical trials are needed to provide a comparative evaluation of this technique for the treatment of pediatric RHCC.

## Conclusion

Our single-center experience suggests the safety and feasibility of percutaneous US-guided RFA for pediatric RHCC. Further investigation in large-scale randomized clinical trials is necessary to determine the role of RFA in pediatric RHCC.

## Data Availability

The datasets generated and/or analyzed during the current study are not publicly available, but are available from the corresponding author on reasonable request.
